# Separation and pain perception of Elastomeric, Kesling and Kansal separators

**DOI:** 10.1590/2177-6709.24.2.042-048.oar

**Published:** 2019

**Authors:** Tulika Tripathi, Navneet Singh, Priyank Rai, Neha Khanna

**Affiliations:** 1 Maulana Azad Institute of Dental Sciences, Department of Orthodontics and Dentofacial Orthopedics (New Delhi, India).

**Keywords:** Bauschinger effect, Banding, Fixed appliance, VAS

## Abstract

**Introduction:**

Various types of separators have been advocated, but the ideal separator should produce optimum separation with minimal pain and discomfort.

**Objective:**

The objective of this study was to evaluate and compare the amount of separation achieved by three different types of separators (Elastomeric, Kesling and Kansal), and to assess the associated pain and discomfort.

**Methods:**

A random single-blind split-mouth study was conducted on 108 patients seeking fixed orthodontic treatment, in which two different separators were used on each side in both the arches for a single patient. After five days, the amount of separation was measured with a feeler gauge. Visual Analogue Scale (VAS) scoring was performed by the patient on each day, to evaluate pain perception. Discomfort was evaluated by questionnaire filled by the patient at the time of separator removal.

**Results:**

The greatest amount of separation was seen with the elastomeric separators, while the smallest separation was seen with Kansal separators. VAS scoring showed maximum pain at day 1 with all the three separator types. Highest pain was perceived in the Elastomeric separators group, followed by Kesling and Kansal separators, respectively. Statistically significant difference was found in VAS score of Elastomeric separators, when compared to both Kesling and Kansal, on day 1 and 2 (*p*= 0.001). Analysis of the questionnaires revealed that a greater number of patients experienced discomfort with elastomeric separators placement (69.4%), which was statistically significant (*p*< 0.01) when compared to the other two types of separators. Answers to the other questions were comparable, except for the need for medications, which was reportedly highest with elastomeric separators.

**Conclusion:**

Kesling separators produce adequate separation with minimal discomfort and pain, compared to Elastomeric and Kansal separators.

## Resumo

**Introdução:**

diversos tipos de separadores de dentes já foram descritos e seu uso, justificado na literatura. Porém, o separador ideal deve produzir uma força adequada para realizar a separação com o mínimo de dor e desconforto.

**Objetivo:**

o objetivo do presente estudo foi avaliar e comparar a separação ortodôntica obtida por meio de três diferentes tipos de separadores (Elastômeros, Kesling e Kansal), bem como a dor e o desconforto a eles vinculados.

**Métodos:**

foi conduzido um estudo randomizado, cego e de boca dividida em 108 pacientes interessados em realizar tratamento ortodôntico, nos quais foram utilizados dois separadores diferentes, um em cada lado de ambas as arcadas de cada paciente. Cinco dias após a inserção dos dispositivos, a quantidade de separação foi mensurada com o auxílio de um medidor (calibrador de folga). Para avaliar a dor, os pacientes preencheram uma Escala Visual Analógica (EVA) a cada dia do estudo; e, para a avaliação do desconforto, preencheram um questionário no dia da remoção dos separadores.

**Resultados:**

os Elastômeros obtiveram a maior quantidade de separação, enquanto os separadores Kansal conseguiram a menor separação. A análise das EVAs apontou a maior intensidade de dor no primeiro dia, para os três tipos de separadores, sendo os maiores índices relatados no grupo de Elastômeros, seguido pelos grupos Kesling e Kansal, respectivamente. Diferenças estatisticamente significativas foram encontradas nas escalas EVA dos Elastômeros em comparação aos separadores Kesling e Kandal, nos dias 1 e 2 (*p*= 0,001). A análise dos questionários mostrou que um grande número de pacientes relatou desconforto após a inserção dos Elastômeros (69,4%), valor estatisticamente significativo em comparação aos outros dois tipos de separadores (*p*< 0,01). As respostas às outras questões foram semelhantes entre os grupos, com exceção da necessidade de uso de medicamentos, a qual foi maior no grupo dos Elastômeros.

**Conclusão:**

os separadores Kesling produziram uma separação adequada, com o mínimo de dor e desconforto, em comparação aos Elastômeros e separadores Kansal.

## INTRODUCTION

Placement of orthodontic bands requires separation between adjacent teeth, due to the presence of tight interproximal contacts. Band placement following improper separation may lead to hyalinization of periodontal ligament and evokes pain response of mechanoreceptors. The average thickness of orthodontic band is 0.16 mm,^1^ ^-^ ^3^ which requires a separation of 0.25mm.^4^ ^,^ ^5^ In contemporary Orthodontics, various methods for separation - like brass wire, elastic ring separator, Kesling separator, C separator, dumbbell shaped separator, NiTi spring separator, Kansal separator, etc. - have been used.^6^ The major drawback of commonly available separators is that they get dislodged from their position once the space is created, and may be ingested or get wedged between adjacent teeth, causing acute localized periodontitis.^7^ Separators should be easy to place with little or no discomfort, easily cleaned, radio-opaque^8^ and not be lost or dislodged.^9^

The placement of separators causes pressure, tension, soreness and pain, which can be detrimental to the patient’s attitude towards further orthodontic procedures.^10^ ^-^ ^12^ The separator-associated pain also interferes with functions like chewing or dietary pattern, leading to discomfort and need for medication. A subjective method for pain assessment is the Visual Analogue Scale (VAS), which has been extensively used in various studies for assessment of pain intensity.^9^ ^,^ ^13^ ^,^ ^14^ On the other hand, an objective method for assessment of separator-associated pain is the use of questionnaires to evaluate patient’s discomfort. Thus, an ideal separator in orthodontic speciality is the one that creates optimum separation with minimal pain and discomfort. Hence, the current study was developed to evaluate and compare the amount of separation achieved by three different types of separators (Elastomeric, Kesling and Kansal), and to assess the associated pain and discomfort.

## MATERIAL AND METHODS 

**Figure 1:**
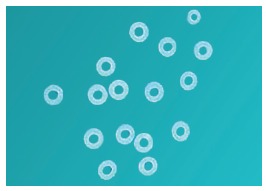
Elastomeric separator (Ortho Organizers).

**Figure 2:**
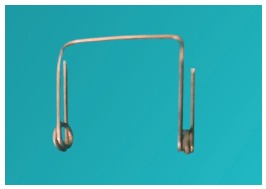
Kansal separator.

**Figure 3:**
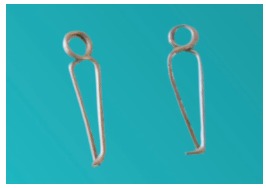
Kesling separator.

The study was approved by the Ethical Committee of Maulana Azad Institute of Dental Sciences and was conducted on 108 patients (54 male and 54 female, mean age of 17.12±3.05 years) seeking fixed orthodontic treatment in the aforementioned institution. The sample size calculation was based on an alpha significance level of 5% (0.05) and beta of 20% (0.20), to achieve 80% power test to detect a mean difference of 0.1 mm with pooled standard deviation of 0.09 mm for separation. Although results showed that each group should comprise 13 individuals, 36 patients were enrolled in each of the three groups, to compensate for any attrition bias. The selected patients had no history of previous orthodontic treatment and presented all permanent teeth in both the arches, except third molars. An informed consent was obtained from all the patients. Three types of separators (Elastomeric separators, Ortho Organizers - Fig 1; Kesling separators - Fig 2; and Kansal separators - Fig 3) were used for separation before placement of orthodontic bands. 

**Figure 4:**
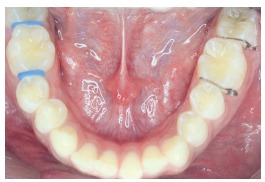
Intraoral photograph of the patient using Elastomeric separator on one side and Kesling separator on the other (Group 1).

**Figure 5:**
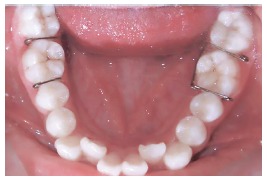
Intraoral photograph of the patient using Kesling separator on one side and Kansal separator on the other (Group 2).

**Figure 6:**
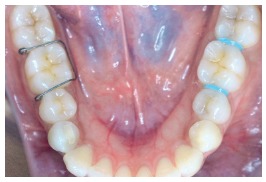
Intraoral photograph of the patient using Elastomeric separator on one side and Kansal separator on the other (Group 3).

This was a single-blind, split-mouth study in which two different separators were used on each side in both the arches for a single patient, and the type of separator was randomly selected for each side in every patient. Thus, Group 1 used Elastomeric separator on one side and Kesling separator on the other (Fig 4); Group 2 used Kesling separator on one side and Kansal separator on the other (Fig 5); and Group 3 used Elastomeric separator on one side and Kansal separator on the other (Fig 6). Hence, each type of separator was placed in 72 sites, with a total of 216 sites (108 maxillary and 108 mandibular sites) in 108 patients. 

Commercially available elastomeric separators were placed with the help of separator placing plier. Both Kesling and Kansal separators were made by the same operator. Kesling separator consisted of helix, occlusal and gingival arms, made of 0.016-in Australian wire (Fig 2), and two separate springs, inserted into mesial and distal contacts of the first molar. 

Kansal separator^15^ was fabricated using 0.016-in Australian wire by bending mesial and distal helices, which were joined by a stabilizing wire lying along the lingual surface of the first molar (Fig 3). The separator was engaged from buccal aspect using bird beak plier, and the connecting wire was pulled lingually. 

Patients were informed about the possibility of pain and discomfort in the days following separator placement. They were instructed to take over the pain reliever medication (400mg Ibuprofen) as needed. All the data regarding the separation achieved and pain perception was recorded following the protocol. The recall visit for removal of separators was scheduled 5 days after separator placement. This was based on a previous study claiming the complete disappearance of pain after 5 days of separator placement, along with adequate separation for band placement.^9^

### Measurement of separation achieved 

**Figure 7:**
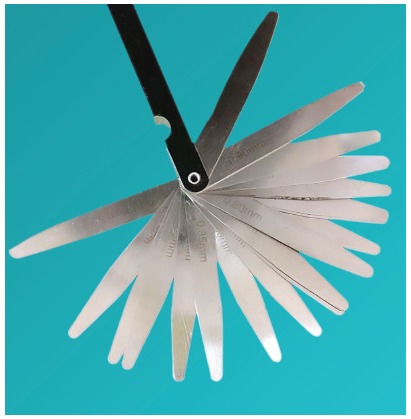
Feeler gauge to measure the amount of separation at teeth contact point.

The amount of separation achieved at each contact point was measured with a feeler gauge at the time of separator removal (Fig 7). The amount of separation was assessed by two different examiners in all the patients, and Kappa statistics were applied to ascertain the reproducibility of results.

### Evaluation of patient’s pain perception and discomfort

The patient’s perception of pain was recorded by means of Visual Analogue Scale (VAS) and a questionnaire. VAS is a simple method to describe pain perceived by the patient.^16^ In this method, a scale of 1 to 10 is drawn, ranging from no pain to most severe pain. The patients were asked to draw the VAS scale on a new paper on each day, and mark a point on this scale, representing the severity of perceived pain on the respective day, until the separator removal. Every day the VAS score was enclosed in an envelope and sealed by the patient, to eliminate any bias due to prior scoring. The numerical value marked by the patient was taken as the VAS score for the respective day.

The questionnaire comprised a series of five questions, of which the first question was asked at the time of separator placement. The remaining four questions were asked at the time of separator removal.

The questions were as follows:

» Q1) Did you experience discomfort during placement of separator? If yes, on which side?
» Q2) Did you experience pain during chewing of food? If yes, on which side?
» Q3) Did you modify dietary pattern?
» Q4) Did you change your mastication site? If yes, on which side?
» Q5) Did you take any medication?

The scoring was done on the basis of number of answers marked as Yes or No.

### Statistical analysis

Statistical analysis was performed using SPSS (Statistical Package for the Social Sciences) for Windows (version 15.0). Categorical variables were described as frequency (percentage); mean ± standard deviation was used for continuous parameters. Differences between groups were compared by the Student *t* test and ANOVA (with *post-hoc* Dunnett T test; *p*-values < 0.05 were regarded as statistically significant. 

## RESULTS

### Separation effects

The inter-examiner reliability for assessment of the amount of separation was found to be good, as shown by Kappa statistics of more than 0.7. The greatest amount of separation was seen with the elastomeric separator (0.45 mm), while the smallest separation was observed with Kansal separator (0.22 mm) (Table 1), and both were statistically significant (*p*= 0.0001). 

**Table 1:** Mean of separation achieved by the three separators.

	Elastomeric separator (n = 72)	Kesling separator (n = 72)	Kansal separator (n = 72)	ANOVA test	Elastomeric separator vs Kesling separator	Kesling separator vs Kansal separator	Elastomeric separator vs Kansal separator
	Mean ± SD (mm)	Mean ± SD (mm)	Mean ± SD (mm)	(p-value)	(p-value)	(p-value)	(p-value)
MxMC	0.45 ± 0.07	0.30 ± 0.05	0.22 ± 0.05	0.001**	0.001**	0.001**	0.001**
MxDC	0.44 ± 0.07	0.30 ± 0.03	0.22 ± 0.04	0.001**	0.001**	0.001**	0.001**
MdMC	0.44 ± 0.05	0.30 ± 0.02	0.22 ± 0.04	0.001**	0.001**	0.001**	0.001**
MdDC	0.43 ± 0.06	0.30 ± 0.03	0.20 ± 0.03	0.001**	0.001**	0.001**	0.001**

**p < 0.001 = highly significant. * p < 0.01 = significant. Significance level was set at p < 0.05.

MxMC = Maxillary mesial contact, MxDC = Maxillary distal contact, MdMC = Mandibular mesial contact, MdDC = Mandibular distal contact.

### Pain and discomfort 

All the 108 patients completed the study and the response rate was excellent, since all the patients answered the questionnaire and marked the VAS scores on each day. VAS scoring revealed maximum pain at day 1 with the three types of separators. Highest pain was perceived in the Elastomeric separators group, followed by Kesling and Kansal separators (Table 2). The intergroup comparison revealed statistically significant difference in VAS score of Elastomeric separators, compared to both Kesling and Kansal separators on day 1 and 2 (*p*= 0.001). The intragroup comparison of VAS score showed statistically significant reduction of pain with Elastomeric separators during all five days of the study, while Kesling and Kansal separators showed significant reduction of pain in the first three days of separator placement (*p*= 0.001) (Table 3). The questionnaire analysis revealed that greater number of patients experienced discomfort with Elastomeric separators placement (69.4%), which was statistically significant (*p*= 0.009) when compared to other two types of separators (Table 4). Answers to the other questions were comparable, except for the need for medications, which was reportedly highest with Elastomeric separators (63.8%).

**Table 2:** VAS scores for different separators from day 1 to day 5.

	Elastomeric separator (n = 72)	Kesling separator (n = 72)	Kansal separator (n = 72)	ANOVA test	Elastomeric separator vs Kesling separator	Kesling separator vs Kansal separator	Elastomeric separator vs Kansal separator
VAS 1	5.11 ± 1.61	3.00 ± 1.62	2.89 ± 1.34	0.001**	0.001**	0.98	0.001*
VAS 2	4.06 ± 1.35	2.33 ± 1.17	2.28 ± 1.21	0.001**	0.001**	0.96	0.001**
VAS 3	1.67 ± 0.86	1.17 ± 0.91	1.11 ± 0.74	0.01*	0.57	0.98	0.14
VAS 4	1.28 ± 0.56	1.17 ± 0.69	1.00 ± 0.75	0.22	0.84	0.70	0.22
VAS 5	1.08 ± 0.43	1.06 ± 0.63	0.83 ± 0.60	0.12	0.99	0.34	0.14

**p < 0.001 = highly significant. * p < 0.01 = significant. *Significance level was set at p < 0.05.

**Table 3:** Intragroup comparison of VAS score from day 1 to day 5.

	Elastomeric	Kesling	Kansal
VAS 1 *vs* VAS 2	0.001**	0.001**	0.001**
VAS 2 *vs* VAS 3	0.001**	0.001**	0.001**
VAS 3 *vs* VAS 4	0.001**	1.00	0.53
VAS 4 *vs* VAS 5	0.006**	0.04	0.12

**p < 0.001 = highly significant. * p < 0.01 = significant. *Significance level was set at p < 0.05.

**Table 4:** Percentage of affirmative answers to the questionnaire.

Question	Elastomeric separator (n = 72)	Kesling separator (n = 72)	Kansal separator (n = 72)	p value
Q1) Did you experience discomfort during placement of separator? If yes, on which side?	50 (69.4%)	24 (33.3%)	40(55.5%)	0.009*
Q2) Did you experience pain during chewing of food? If yes, on which side?	38(52.7%)	26(36.1%)	22(30.5%)	0.137
Q3) Did you modify dietary pattern?	30(41.6%)	22(30.5%)	16(22.2%)	0.207
Q4) Did you change your mastication site? If yes, on which side?	30(41.6%)	16(22.2%)	16(22.2%)	0.111
Q5) Did you take any medication?	46 (63.8%)	22(30.5%)	22(30.5%)	0.004*

**p < 0.001 = highly significant. * p < 0.01 = significant. *Significance level was set at p < 0.05.

## DISCUSSION

Fixed orthodontic therapy involves the placement of bands and brackets as a medium to apply the necessary forces. The procedure of banding the molars requires adequate separation, to facilitate band placement without any undesirable periodontal injury. The earliest attempts for tooth separation were done by Angle^17^ and Case,^18^ who used brass wire and separating tape, respectively.^10^ Currently, two major types of separators, namely Elastomeric and spring separators, are in use.^19^ Elastomeric separators are most commonly used due to the easy availability and convenient use.^6^ However, studies have shown that elastomeric separators are frequently dislodged^20^ and cause greater amount of pain and discomfort.^9^ ^,^ ^21^ But in the present study all the separators, including Elastomeric, were retained until the end of day 5.

Another limitation of the conventional elastomeric separator is the difficulty for placement around tight contacts. For such cases, Kesling separator has been advocated, which can be more easily placed.^22^ One of the most recent spring separators has been developed by Kansal et al,^15^ which can be placed around tight contacts and is claimed to achieve adequate separation with good retention.

### Measurement of separation

The maximum separation was observed with Elastomeric separator, which was statistically significant, followed by the Kesling and Kansal separators, respectively. This finding was in concordance with the results of previous studies.^9,22^ However, according to previous studies,^4^ ^,^ ^5^ the optimum amount of separation for band placement would be 0.25 mm at the contact area. Results of the present study showed that Elastomeric separators created excessive separation (0.45 mm), while the Kansal separator showed inadequate separation (0.22 mm); on the other hand, the separation produced by the Kesling separators was found to be adequate (0.30 mm).

Although both Kesling and Kansal separators use the principle of spring action, the difference in separation achieved with both the spring separators may be attributed to the differences in their designs. The Kesling separator comprises of two free arms and a helix with two and half coils, which results in greater flexibility. On the other hand, the Kansal separator has unified arms and a helix with one and a half coil. In addition to the design features, the working of Kesling separator is more advantageous as it works on the principle of closed coil spring, which has the advantage of showing reverse Bauschinger effect, wherein the activation is done in the same direction of the coil. In contrast, the Kansal separator shows Bauschinger effect, wherein the activation is done opposite to the direction of coil, which results in reduction of yield strength. 

### Pain and discomfort

Separator placement is the first step in orthodontic therapy, with the goal of achieving adequate separation for band placement. However, the resulting pain due to its placement can affect the motivation of the patient towards the further orthodontic treatment. To evaluate this pain, VAS scale was used in each patient, due to its simplicity and proven ability to distinguish between intraoral pain on either side.^9^ ^,^ ^23^ ^,^ ^24^ In addition, the discomfort experienced in performing normal oral functions during the period of separation was evaluated qualitatively by means of a questionnaire.

The amount of pain was found to be greater during the first 24 hours of separator placement, similar to previous studies.^9^ ^,^ ^12^ ^,^ ^24^ ^,^ ^25^ The intragroup comparison revealed that the pain with elastomeric separator was so intense at the time of placement that the pain reduction on each day, for the entire research period, was statistically significant. On the other hand, Kesling and Kansal separators showed significant pain reduction only until day 3, which means that lesser residual pain persists after the third day of placement, leading to better patient comfort.

The highest pain perception was seen with elastomeric separator which was statistically significant as compared to the other 2 methods on day 1 and 2. This difference in pain can be due to the excessive separation brought about by the Elastomeric separators. The least amount of pain was produced by the Kansal separators, which may correspond to the least amount of separation achieved. Answers to the questionnaires revealed that greater discomfort occurred with Elastomeric separators, which may have happened because it traverses through the contact point, while the other two springs require gingival and occlusal approach for placement. Similar findings were seen in previous studies.^9^ ^,^ ^22^

The greater amount of discomfort seen with Elastomeric separators was found to affect functions like choice of masticatory site, dietary pattern and need for medication. However, the other two methods of separation were comparable in this regard. Besides the questionnaire, an additional complaint of tongue irritation in case of Kansal separators was recorded.

### Limitation of the study

The contact point morphology, tightness of contact and pain threshold vary from patient to patient. Hence, the result of the present study and effectiveness of any individual separator with regard to separation and pain may not be applicable to every patient. 

### Future scope

Further studies for evaluation of separation effects of different types of separators may be conducted taking into account other factors like gender, periodontal status and dietary pattern, for more objective assessment.

## CONCLUSION

The Kesling separator produces adequate separation with minimal discomfort and pain, compared to Elastomeric and Kansal separators. 
